# Bacterial Lysates as Immunotherapies for Respiratory Infections: Methods of Preparation

**DOI:** 10.3389/fbioe.2020.00545

**Published:** 2020-06-05

**Authors:** Norma Suárez, Florencia Ferrara, Analia Rial, Valerie Dee, Jose A. Chabalgoity

**Affiliations:** Departamento de Desarrollo Biotecnológico, Instituto de Higiene, Facultad de Medicina, Universidad de la República, Montevideo, Uruguay

**Keywords:** respiratory tract infections (RTIs), immunomodulator, bacterial lysates, mechanical, alkaline, bacteriophage, vaccine

## Abstract

Bacterial lysates, prepared from the microorganisms most frequently involved in human Respiratory Tract Infections (RTIs) have been in the market for several decades, and at present, several different brands are available in many countries worldwide. They all claimed to exert local and systemic immunomodulatory effects but different clinical trials show disparate results between them. The lack of consistency of predicted therapeutic effects has undermined their clinical use and hampered licensing in several countries. One explanation for such lack of consistency in the results is that their methods of preparation are also very different. Here, we review the available literature describing methods of preparation of bacterial lysates, including patent disclosure documents. We found a great variety of methodologies of preparation and a lack of standardized procedures among them. The main conclusion of our study is that there is a clear need for standardized protocols of production to obtain comparable results in clinical trials worldwide.

## Introduction

Respiratory tract infections (RTIs) such as acute bronchitis, community-acquired pneumonia (CAP), and others are the most prevalent infectious diseases in humans and cause millions of deaths annually worldwide (World Health Organization, 2017). Further, respiratory infections are the main comorbidity factor in chronic obstructive pulmonary disease (COPD). Although in the last couple of decades there have been significant improvements in treatment and control of the burden of these infections, there is still a lack of vaccines against most of the infectious agents responsible for RTIs; so other prophylactic strategies must be developed (Cazzola et al., 2008; Esposito et al., 2018). One strategy for treating these infections is the use of bacterial lysates (BLs) which were introduced in the 1970s as oral vaccines for the prevention and treatment of RTIs (Cazzola et al., 2012a; Hancock et al., 2012; Esposito et al., 2018). These lysates are mixtures of antigens derived from inactivated pathogens frequently involved in RTIs.

Over the last few decades, BLs have gained renewed attention because of their contribution to the reduction of recurrent RTIs in childhood (Gutiérrez-Tarango and Berber, 2001; Rozy and Chorostowska-Wynimko, 2008; Navarro et al., 2011). Positive outcomes have also been reported for treatment of chronic obstructive pulmonary disease (COPD) in adults (Cazzola et al., 2012b; Kearney et al., 2015). Studies have shown that BLs are effective immunostimulators, triggering specific responses in local areas of the mucosal immune system (Braido et al., 2007, 2011; Cazzola et al., 2012b; Kearney et al., 2015).

Despite extensive clinical use of BLs, their effects on the immune system are only partially known. The evidence from most studies suggests that BLs act as immunomodulators capable of inducing antibodies against specific pathogens as well as immunoregulatory responses at mucosal tissues (Braido et al., 2011; Cazzola et al., 2012b; Kearney et al., 2015; Esposito et al., 2018; Triantafillou et al., 2019). Particularly, it has been demonstrated that they can interact with different cells through bacterial wall components, such as proteoglycans or lipopolysaccharides, which interact with Toll-like receptors (TLR) on monocytes/macrophages, dendritic, or epithelial cells. These interactions stimulate the differentiation of monocytes into macrophages and activate immature dendritic cells, resulting in the production of selected chemokines and cytokines. Altogether, these responses will induce the recruitment of innate effector cells to the mucosal sites and induce lymphocyte activation that could help in the protection against invading pathogens. Some authors suggest that these responses do generate a state of “pre-alert” against infection (Kearney et al., 2015).

The lack of standardized protocols might be a barrier to the experimental reproducibility of immunostimulatory effects. Different authors have worked with different microorganism cell fractions as components of BLs. In particular, the various methods by which the lysates were prepared may have contributed to the inconsistencies between their reported biological effects. Possibly lack of rigor in experimental design, insufficient patient numbers, or other technical faults have led to some level of mistrust in the clinical trials performed (Cazzola et al., 2008). Standardized production protocols would be a useful step toward overcoming some of the current difficulties comparing the biological effects of BLs.

The two most common methods used for bacterial lysis are alkaline treatment and mechanical disruption, although heat or detergents have also been used (Bauer et al., 2008; Braido et al., 2011; Cazzola et al., 2012a). Alkaline lysis may produce protein denaturation of bacterial antigens, whereas mechanical disruption does not supposedly alter the antigenic structures in the BLs (Kearney et al., 2015; Jurkiewicz and Zielnik-Jurkiewicz, 2018; Triantafillou et al., 2019). Each bacterial strain is grown independently, harvested, inactivated by the selected procedure and then optionally lyophilized. Individual lysates are mixed in fixed proportions to give a polyvalent BL.

Commercial polyvalent bacterial lysates are available in the form of oral capsules or sublingual tablets (Table 1; European Medicines Agency, 2019). Among the best known, are Broncho-Vaxom® as an example of a bacterial immunostimulant obtained by chemical lysis and Ismigen® or Ribomunyl-D 53 composed of unaltered antigenic particles obtained by mechanical cell disruption (Braido et al., 2007; Cazzola et al., 2008). Other BLs commercially available are in use for treatment of other infections, such as urinary tract infections (Huber et al., 2000; Bauer et al., 2002, 2015; Aziminia et al., 2019; Wawrysiuk et al., 2019) or for tuberculosis relapse (Dyachyk, 2006), also with good results.

**Table 1:** Characteristics and clinical outcomes of commercial bacterial lysates used for treating RTIs.

**Bacterial lysate**	**Bacterial composition**	**Preparation method**	**Administration /Formulation**	**Clinical outcomes**
Broncho-Vaxom (OM-85 BV)	*S. aureus*, *S. pneumoniae*, *S. pyogenes*, *S. viridans*, *M. catarrhalis*, *H. influenzae*, *K. pneumoniae*, *K. ozaenae*	Alkaline lysis	Oral capsules	In pediatrics: Reduction in the incidence rate of RTIs (Jara-Pérez and Berber, 2000; Gutiérrez-Tarango and Berber, 2001; Schaad, 2010; Esposito et al., 2014). Reduction of the duration of RTIs (Jara-Pérez and Berber, 2000; Gutiérrez-Tarango and Berber, 2001). Reduction of antibiotic requirement (Jara-Pérez and Berber, 2000; Gutiérrez-Tarango and Berber, 2001; Esposito et al., 2014; Chen et al., 2017). Reduction of the frequency of rhinosinusitis attacks (Chen et al., 2017). In patients with COPD: Reduction of the frequency of exacerbations (Solèr et al., 2007; Tang et al., 2015). In HIV+ patients: Reduction in the incidence rate of RTIs and in the use of antibiotics (Capetti et al., 2013).
Liuvac LW50020	*S. aureus*, *S. pneumoniae*, *S. pyogenes*, *S. mitis*, *M. catarrhalis*, *H. influenzae*, *K. pneumoniae*	Alkaline lysis	Oral tablets	In pediatrics: Reduction in the incidence rate and duration of RTIs and antibiotic requirement (Ruah et al., 2001). In adults: - Reduction in the number and duration of RTIs and antibiotic requirement (Grevers et al., 2000). - Reduction of bacterial count in colonized patients (Zagólski et al., 2015).
Ismigen PBML	*S. aureus*, *S. pneumoniae*, *S. pyogenes*, *S. viridans*, *M. catarrhalis*, *H. influenzae*, *K. pneumoniae*, *K. ozaenae*	Mechanical lysis	Sublingual tablets	In pediatrics: Reduction in the incidence rate of RTIs (Rosaschino and Cattaneo, 2004). In patients with COPD: Reduction the number of days with fever, hospitalization (Braido et al., 2015), reduction in number and intensity of exacerbation (Ricci et al., 2014; Braido et al., 2015).
Lantigen B	*S. aureus*, *K. pneumoniae*, *S. pneumoniae*, *H. influenzae*, *M. catarrhalis*, *S. pyogenes*	Alkaline lysis	Oral drops	Reduction in the number of acute episodes in patients with recurrent RTIs (Braido et al., 2014).
Ribomunyl D53	*K. pneumoniae*, *S. pneumoniae*, *S. pyogenes*, *H. influenzae*	Proteoglycans and ribosomes	Oral tablets or granules	In pediatrics: Reduction of the duration of the infectious episodes (Mora et al., 2002; Fiocchi et al., 2012). The reduction incidence rate of RTIs (Mora et al., 2002).

## Types of Bacterial Lysates

### Alkaline Bacterial Lysates

Alkaline lysis uses sodium hydroxide ions to disrupt the cell membrane structure by changing pH to the range of 11.5–12.5.

One of the first bacterial lysates obtained by alkaline extraction was Lantigen B whose *in vivo* effects were first described in the 1970s (Tyrrell et al., 1972) and more recently demonstrated by Braido et al. (2014). Later on, Bauer et al. (1995) patented another process that included cultivation of *Escherichia coli* in an aqueous medium followed by alkaline extraction of bacterial proteins, in the presence of a “diluted aqueous source of OH- ions.” The alkaline lysis process used included concentration, ultrafiltration, diafiltration of the lysate, and lyophilization. The authors further described the extraction of lipopolysacharides (LPS) by ion-exchange chromatography and characterization of the lysate by biochemical analyses of amino acids racemized during the alkaline extraction. Additionally, other lysis processes for bacteria causing RTIs were included in the patent with variation in sodium hydroxide (NaOH) concentrations (Table 2; Bauer et al., 1995).

**Table 2:** Methods of bacterial lysates preparation.

**Lysis method**	**Bacterial composition**	**Lysis** **(Time h)**	**Lysis** **(Temp)**	**Preparation techniques used**	**Biochemical analysis of the lysate**	**References**
Alkaline NaOH (conc) 0.01–1%	*E. coli*	Several	30 to 45°C	Ultrafiltration Diafiltration	LPS extraction (Triton X-114) Ion exchange Chromatography Amino acids racemization	Bauer et al., 1995
Alkaline NaOH (conc) 0.1–1.2 N	*S. aureus, M*. *catarrhalis, K*. *pneumoniae, S*. *pneumoniae, H*. *influenzae*	20 to 40	30 to 60°C	Centrifugation Filtration Ultrafiltration	Proteins, Glutamic acid and LPS measurements	Bauer et al., 2008
Mechanical Ultrasonic treatment	*S. aureus, M*. *catarrhalis, K*. *pneumoniae, S*. *pneumoniae, H*. *influenzae*	Variable	55 to 65°C	Liophilization	Optical density determination	Illiarten, 1971
Mecanical High pressure valve	*S. aureus, M*. *catarrhalis, K*. *pneumoniae, S*. *pneumoniae, H*. *influenzae*	No mention	No mention	Washing, Filtration, Centrifugation, Liophilization	Gram technique analysis Rocket electrophoresis	Melioli and Fasani, 2004
Mecanical Sonication	*S. pneumoniae,* * M. catarrhalis, K*. *pneumoniae*, *Micrococcus* spp*, H. influenzae,* *Streptococcus* spp. (i.e., *S. anhemoliticus* and *S. viridans*).	26	Cycles Heat at 70°, keep at 4° Additional cycle 70°C	No mention Partially private preparation	No mention	Coviello et al., 2014
Ribosomal lysatesHigh pressure homogenizer	*S.pneumoniae, S.* * pyogenes,* * K.pneumoniae,* * H.influenzae,* * proteoglycan of K.* * pneumoniae*	-	Low Temp	Centrifugation Filtration, sterilization	No mention	Michel et al., 1978
Bacteriophages	*S. aureus, K.* * pneumoniae,* *P. aeruginosa*.	3–48 h incubation		Filtration, liophilization	SDS Gel electrophoresis	Pillich and Balcarek, 2011

Bauer and other collaborators patented years later a bacterial extract for respiratory infections with a brief description of the process for its preparation. This was an alkaline lysate extracted from bacterial strains of *Staphylococcus aureus, Moraxella catarrhalis, Klebsiella pneumoniae, Streptococcus pneumoniae*, and *Haemophilus influenzae* (Bauer et al., 2008, 2010). The lysing process described in the patent covered a range of diverse possible conditions at the different stages of the process from bacteria fermentation to the resulting modified protein mixture. According to the authors, alkaline lysis of each strain or set of strains is suitable for all bacteria causing RTIs.

Among the commercially available formulations prepared by alkaline lysis are OM-85 BV (Broncho-Vaxom®) and Liuvac (LW-50020). OM-85 BV (Broncho-Vaxom®) is a mixture of *H. influenzae, S. pneumoniae, K. pneumoniae, Klebsiella ozaenae, S. aureus, Streptococcus pyogenes, Streptococcus viridans*, and *M. catarrhalis*. Liuvac is a mixture of bacterial lysates from *S. aureus, S. pneumoniae, S. pyogenes, K. pneumoniae, M. catarrhalis*, and *H. influenzae* that has been evaluated also for the treatment of chronic bronchitis and COPD (Cazzola et al., 2008, 2012b; Esposito et al., 2018).

For trademarked bacterial lysate formulations like OM-85 BV (Broncho-vaxom®) there is no access to a detailed preparation protocol stipulating, for example, NaOH concentrations, temperatures, or periods used for the cell lysis process.

### Mechanical Bacterial Lysates

The most widely used mechanical methods of bacterial cell lysis are ultra-sonication and high-pressure homogenization. For homogenization, different pressures are used depending on the cell type (Goldberg, 2008).

One of the first patents for mechanical bacterial lysate preparation found in the literature reported the use of desiccated and partially lysed bacterial antigens (Illiarten, 1971). The author described lysis of *S. aureus, K. pneumoniae, Haemophilus influenzae, S. pneumoniae*, and *M. catarrhalis*, by slow freezing followed by rapid thawing of the cell suspension at different temperatures ranges with ultrasonic treatment for different periods of time (Table 2).

Melioli and Fasani patented a mechanically lysed preparation of *S. aureus, K. pneumoniae, S. pneumoniae, Haemophilus influenzae*, and *M. catarrhalis*, separately or in combination (Melioli and Fasani, 2004). The particulate bacterial lysates were obtained by fragmentation of bacterial cells using a high-pressure valve, followed by separation of the unaltered antigenic particles fraction from soluble components by centrifugation, washing, and filtration (Table 2).

Later on, Coviello et al. (2014) described mechanically prepared lysates from *S. pneumoniae, M.catarrhalis, K. pneumoniae, Micrococcus* spp, *Haemophilus influenza*, and *Streptoccocus* spp. (i.e., *Streptococcus anhemoliticus* and *S. viridans*) by using sonication to disrupt the bacterial cell walls. In their particular lysis process, the bacterial material underwent cycles of temperature changes before the lysate was analyzed to confirm that no viable organisms remained (Table 2; Coviello et al., 2014).

The largest marketed representative brand of mechanical lysates preparations is Ismigen® which is a polyvalent mechanical bacterial lysate (PMBL) composed of lysates from *S. aureus, S. pyogenes, S. viridans, K. ozaenae, H. influenzae* type b*, M. catarrhalis*, and *S. pneumoniae*.

Another type of mechanical lysates are ribosomal-rich lysates. They are obtained by disrupting the cells with micro glass beads in a homogenizer, a technique first described in 1965 (Youmans and Youmans, 1965). Those authors showed that the ribosomal fraction obtained from ruptured myco-bacteria exhibited high immunogenicity. Since then, various authors have studied the protection conferred in animals using ribosomal fractions from bacteria such as *S. pneumoniae, Salmonella* Typhimurium, and *Neisseria meningitidis* (Thompson and Snyder, 1971; Venneman and Berry, 1971; Thomas and Weiss, 1972; Swendsen and Johnson, 1976). Then, Michel et al. (1978) described a ribosome preparation extracted from bacterial biomass after bacterial fermentation followed by mechanical disruption under pressure at low temperature, a series of centrifugations to remove unbroken cells and several filtration and sterilization steps to obtain the bacterial lysate (Table 2; Michel et al., 1978). Later, Dussourd d'Hinterland and colleagues marketed an intranasal polyvalent ribosomal vaccine for humans named Ribomunyl-D 53 composed of ribosomal preparations from *K. pneumoniae, S. pneumoniae, S. pyogenes* group A, and *Haemophilus influenzae*, with a membrane proteoglycan of a non-capsular strain of *K. pneumoniae* as an adjuvant (Dussourd d'Hinterland et al., 1980). Several studies have been performed showing its efficacy as immunostimulants (Lauener, 1994; Clot, 1997; Caliot et al., 2000; Bellanti et al., 2003; Bousquet and Fiocchi, 2006). As compared with alkaline bacterial extracts such as OM85 BV, D53 was shown to induce more antibody producing cells in the tonsils of treated children and the authors suggested that partially transcribed proteins present in the ribosomal enriched preparation could contain potent epitopes that would interact with immunocompetent cells more efficiently than large proteins present in the bacterial extract (Béné et al., 1993).

Altogether, mechanical cell disruption has proven to be an efficient method, achieving lysis of 80–100% of the bacteria (Cazzola et al., 2012a). As other authors have previously proposed, mechanical lysates contain particles that maintain several features of intact antigenic molecules, thus providing better interaction with Toll-like receptors which is one of the initial aims of these preparations (Villa et al., 2010; Cazzola et al., 2012a,b; Esposito et al., 2018; Triantafillou et al., 2019). However, the method has the limitation of heat generation which must be minimized to avoid protein denaturalization under the process and it is more expensive than alkaline lysis.

### Bacterial Lysates Preparations by Bacteriophages

Bacterial lysates prepared by infecting bacteria with selected bacteriophages were patented in 2011 by Pillich and Balcarek who had been working with bacterial lysates since 2003 (Pillich and Balcarek, 2011). The initial idea dates back to 1917 when the use of bacteriophages for the treatment of bacterial diseases was proposed by D' Herelle (Fruciano and Bourne, 2007).

The authors prepared bacterial lysates from strains of *S. aureus, K. pneumoniae*, and *Pseudomonas aeruginosa*. They incubated the bacteriophages with the bacterial culture for a certain period. The resulting lysates containing cellular materials such as cell wall components, cellular membranes, proteins, ribosomal fractions, glycoproteins, DNA, and RNA, were then filtered and sterilized. The authors suggested that the use of bacteriophages is effective for all types of bacteria.

Because the protocol is patented the authors did not provide specific details on incubation times or bacteriophages doses. However, an approximation to bacteriophages culture concentration is given as well as approximate incubation times (from 3 to 48 h). The techniques used for antigenic pattern determination are given in Table 2 (Rothbard et al., 2014).

### Clinical Effects

Many clinical studies have been carried out to evaluate the immunostimulatory effect of bacterial lysates, assessed as beneficial effects in patients susceptible to respiratory tract infections (RTIs) as children, elderly or COPD patients. Among all the bacterial lysates described in the literature, the largest number of clinical trials have been performed on OM-85 BV (Broncho-vaxom®) and Ismigen® (PMBL) as examples of chemical or mechanical lysis respectively (Bessler et al., 2010; Navarro et al., 2011; Lanzilli et al., 2013; Tang et al., 2015).

OM-85 has shown good safety and tolerance in many studies with reduction in recurrences of respiratory infections in children and adults (Jara-Pérez and Berber, 2000; Gutiérrez-Tarango and Berber, 2001; Solèr et al., 2007; Schaad, 2010). A meta-analysis of eight randomized controlled trials performed by Schaad et al., showed a reduction in the cases of recurrent RTIs among children treated with OM-85 (Schaad, 2010). Then, another meta-analysis also confirmed that data and showed the protective effect of OM-85 on recurrent RTIs in children (Yin et al., 2018; Esposito et al., 2019). Regarding COPD patients, a meta-analysis published by Pan et al., concluded that current evidence was inadequate for supporting a beneficial effect of OM-85BV for COPD patients, in terms of duration of hospitalization, the severity of acute exacerbation and total adverse events (Pan et al., 2015).

In general, authors agree that there are some limitations among the trials already performed so, there is a need for high-quality, large, multicenter, double-blind, placebo-controlled randomized clinical trials in order to confirm the role of immunostimulants in preventing RTIs in children (Del-Rio-Navarro et al., 2012) as well as adults or COPD patients (Pan et al., 2015).

On the other hand, there are also many clinical studies using BLs prepared by mechanical disruption showing promising results. Rosaschino and Cattaneo (2004) evaluated efficacy and tolerance in pediatric patients treated with PMBL and demonstrated that the treatment was effective with excellent tolerability of the BLs (Rosaschino and Cattaneo, 2004). Braido and Melioli studied another lysate preparation named Lantigen B in patients with recurrent RTIs and demonstrated a significant reduction of the number of acute episodes in these patients (Braido et al., 2014).

Results for the activity of BLs as vaccine have been described as depending partly on the pathogenic strain used, partly (in the case of ribosomal BLs) on the degree of purity of subcellular fraction, and partly on the presence or absence of other cell constituents such as polysaccharides (Michel et al., 1978). Different types of immune responses (cell-mediated or humoral) have been induced in different studies. Administration routes have included nasal, sublingual as well as oral (Schaad, 2010; Cazzola et al., 2012a; Rial et al., 2016).

In summary, several studies have shown the beneficial effects of treatment with bacterial extracts, either obtained through mechanical or chemical procedures. As already mentioned, some authors have stated that mechanical disruption could be a better alternative to chemical lysis due to the preservation of the antigens. However, no study has been performed comparing the biological effects of polyvalent bacterial lysates, prepared from the same bacterial cultures, but using mechanical or chemical lysate procedures (mechanical vs. chemical). This comparative studies could give some light for choosing the best method for preparing immunostimulant lysates.

### Future Outlook

Several methods have been used for years in the preparation of cell lysates (e.g., alkaline treatment or mechanical disruption). Since mechanical disruption is suggested to be the most promising way to lyse the cells, significant technological progress has been made in this field by some companies (Cazzola et al., 2012a; Jurkiewicz and Zielnik-Jurkiewicz, 2018; Esposito et al., 2019). They have developed focused-ultrasonication, an advanced computer-controlled technology that can control the dosage of energy delivered to gently disrupt the cell membranes of mammalian cells or abruptly disrupt the cell walls of bacteria. This technology could address the challenges of standardizing procedures for producing mechanical bacterial lysates (Wenger et al., 2008; Bláha et al., 2017).

## Conclusions

This review summarizes the most prominent publicly available information for production and use of bacterial lysates. Despite their importance in public health and the number of studies done at the clinical level, there is a need for more standardized protocols for the preparation of these bacterial antigens because the literature does not describe lysis procedures in detail or the procedures are not openly available to the research community. The fact that the selected inactivation method is essential for the efficacy of the bacterial lysates is an important aspect that deserves in-depth exploration, especially as some of these treatments can lead to denaturation of the antigens. The lack of standardized protocols leads to different extract performance across laboratories and is a barrier to reproducibility. In turn, this can result in misleading evaluations of the effects produced by these lysates. Standard production protocols would be a useful step toward overcoming some of the current difficulties in comparing the immunological and clinical effects of BLs and their use as immunotherapy for the prevention and treatment of RTIs.

## Author Contributions

NS participated in the conception, information search and assisted in drafting the manuscript. FF and AR participated in the information search, assisted in drafting of the manuscript. VD assisted in language correction. JC assisted in drafting and correction of the manuscript.

## Conflict of Interest

The authors declare that the research was conducted in the absence of any commercial or financial relationships that could be construed as a potential conflict of interest.
